# HIV Prevention Research Experiences Among Men Who Have Sex With Men and Transgender Persons of Color

**DOI:** 10.1007/s40615-022-01340-4

**Published:** 2022-06-09

**Authors:** Aparna Alankar, Jamir Tuten, Travis Love, Jennifer Punsal, Shobha Swaminathan, Amesika N. Nyaku

**Affiliations:** Division of Infectious Diseases, Department of Medicine, Rutgers New Jersey Medical School, Newark, USA

## Abstract

**Purpose:**

Black and Latinx MSM and transgender POC disproportionately experience new HIV diagnoses. Determining effective HIV prevention methods requires the inclusion of these communities in research and thorough post-trial experience evaluations. This study sought to evaluate the experiences of Black and Latinx MSM and transgender POC in HIV prevention research and identify facilitators and barriers to continued trials participation.

**Methods:**

A survey was developed in partnership with the community engagement team based on emerging themes during research participant check-ins with the team. The survey was built in REDCap and distributed to participants via text message. The survey assessed experiences with the research process time commitments, study responsibilities, compensation, experiences with Truvada®, characteristics of the research study team and site, barriers to continued study participation, willingness to participate in future studies, and overall satisfaction. All statistical analysis was completed in Stata.

**Results:**

Forty-four participants were enrolled in the study. Most participants (98%) were satisfied with their experiences in HIV prevention research. Job or school schedules were the most frequently cited barrier to study participation while Truvada® provision and adequate study visit compensation, length, number, and frequency were facilitators. Participants reported that research staff made them feel comfortable when talking about sexual behaviors, alcohol use, mental health, drug use, housing problems, violence in relationships, and legal problems.

**Conclusions:**

Evaluating the experiences of key communities in HIV prevention research can help identify barriers and facilitators to clinical trials engagement and improve the design of future trials.

## Introduction

Black and Latinx men who have sex with men (MSM) and transgender persons of color (POC) are disproportionately affected by HIV in the USA [1]. Despite overrepresentation among new HIV diagnoses, Black and Latinx MSM and transgender POC have relatively low participation in HIV prevention trials [2–4]. A study conducted by the HIV Vaccine Trials Network (HVTN) examined enrollment trends of racial/ethnic minorities in 43 Phase 1 and Phase 2A HIV prevention vaccine clinical trials conducted from 2002 to 2016 in the USA [3]. In comparison to a similar study conducted by Djomand et al. which focused on trials from 1988 to 2002, the HVTN study found that although there were increased percentages of racial/ethnic minorities enrolling in HIV prevention vaccine clinical trials (fluctuating between 17 and 53%), the increase was not proportional to the number of new HIV diagnoses among these groups [3, 4]. Richardson et al. conducted a study of NYC residents who identified as Black MSM and transgender women and found that out of 189 participants, 75% were aware of HIV vaccine trials but only 5.7% had participated themselves and only 9.5% knew someone else who had participated in a HIV vaccine trial, limiting internetwork referrals [2]. Castillo-Mancilla et al. administered a similar survey among adults living with HIV who were attending AIDS Clinical Trials Group-affiliated clinics in the USA and Puerto Rico [5]. Castillo-Mancilla et al. found that compared to White adults, Black or African American and Hispanic adults were “less likely to have been talked to about participating in a study” (76% vs. 67% and 67%, respectively; *p* = 0.001) and more likely to state that “studies were not friendly to their race or ethnicity” (4% vs 17% and 10%, respectively; *p* = 0.001) [5]. Engaging these communities in the clinical trials phase continues to be key to developing acceptable HIV prevention methods. Assessing trial experiences and investigating barriers to clinical trials participation may contribute to increased clinical trials participation. The goal of this study was to investigate barriers, facilitators, and satisfaction with participation for Black and Latinx MSM and transgender POC in HIV prevention studies evaluating novel HIV prevention methods for these communities.

## Methods

### Participants

The study was restricted to cisgender MSM and transgender persons who had participated in HIV prevention clinical research studies conducted at New Jersey Medical School Clinical Research Center (NJMS CRC). All participants were at least 18 years of age. Participants were recruited from the directory of HIV prevention study participants that had consented to be contacted for future research studies. Eligible individuals were contacted via phone or social media by the research team and interested individuals received a text message link to the study specific consent and survey. Each participant had to review and complete the electronic informed consent prior to initiating the survey. The study was approved by the Rutgers University Institutional Review Board.

### Survey

The survey was developed by the study team and collected demographic information such as age, gender, race/ethnicity, and the HIV prevention study in which the respondent participated. Gender was collected with one question asking participants to self-identify their gender as “male,” “transwoman,” “transman,” and “other, please describe.” Specific questions about sexual orientation and sexual behaviors were not asked as the sample of potential participants was taken from parent studies that only enrolled cisgender men or transgender persons that had sex with individuals with a sex assigned at birth of male in the preceding 6 months. For the purposes of the analysis of the survey responses, a participant was categorized as MSM if they were not a transgender person. In order to assess barriers, facilitators, and satisfaction with the research experience, the team created questions based on recurring themes that participants volunteered during their periodic check-in sessions with the community engagement team. The community engagement team was compromised of individuals with shared racial, ethnic, gender, and/or sexual orientation as the study participants and these check-ins frequently occurred as a part of the protocol-defined risk reduction counseling portion of the study visit. Additionally, as needed, ad hoc check-ins were conducted by phone, virtually, and in-person. Questions were constructed to assess experiences with the research process time commitments, study responsibilities, compensation, experiences with Truvada®, characteristics of the research study team and site, barriers to continued study participation, and willingness to participate in future studies. Responses were assessed using a 3-point or 5-point Likert scale and took about 5 min to complete. Questions evaluating overall satisfaction were devised using concepts that emerged after conducting a literature search of other studies evaluating satisfaction with HIV prevention research studies among Black and Latinx MSM and/or transgender persons [6–9]. The survey instrument was pretested with research staff at NJMS CRC for clarity of wording and accessibility on multiple devices, including cell phones, laptops, and tablets. Pilot testing with five participants among the general population was also performed. The study was created and distributed using the REDCap (Research Electronic Data Capture) tool hosted at Rutgers University [10, 11]. REDCap is a secure, web-based software platform designed to support data capture for research studies [10, 11]. The participants were issued reminders at two weeks and one month following initial survey distribution, via text message.

### Statistics

The deidentified dataset was downloaded from REDCap and analyzed in Stata (version 16.1, StataCorp LP, College Station, Texas, USA). Demographic and clinical data was analyzed using descriptive statistics. Continuous data was described using mean and range. Categorical data was described using frequency of count. Pearson’s chi-squared test and Fischer’s exact test were performed to assess statistically significant differences between groups. There were no statistically significant differences by age, gender, race, or study; therefore, the results are reported in aggregate.

## Results

### Attitudes towards HIV prevention trial responsibilities and compensation

Of the cisgender MSM and transgender participants enrolled in HIV prevention studies at our site, 117 participants had consented to be contacted for future research opportunities and all were contacted between March and June 2020. Of the 117 participants contacted, 44 (37.6%) participants completed the survey. The racial/ethnic make-up of study participants was as follows: 20 (48%) Black or African American participants, 10 (24%) Hispanic or Latinx participants, 6 (14%) White participants, 4 (10%) Asian or Pacific Islander participants, and 2 (5%) Native American or American Indian participants. Two participants did not indicate their race. There were 39 cisgender MSM and 5 transgender women enrolled in the study and the mean age was 31 years, ranging from 23 to 54 years.

### Perception of study logistics

**Fig. 1:**
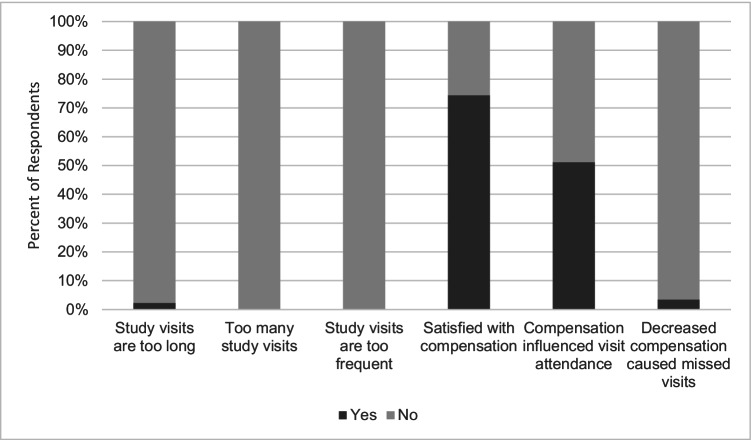
Participants’ perspectives regarding time commitment and compensation for study visits. Participants were asked about the time required to participate in the study and the adequacy of compensation

Participants were asked their satisfaction with the time commitment required to be in the study (Fig. 1). No participants reported that visits were too numerous or too frequent. Forty-three (98%) were satisfied with study visit length. Participant attitudes towards study-specific responsibilities were rated on a 3-point Likert scale (not important, somewhat important, very important). Forty-two (98%) participants reported that completing the study visit was somewhat or very important, all participants reported that communicating with the team was somewhat or very important, all participants reported receiving the study medications was somewhat or very important, and 31 (90%) participants reported taking pills every day was somewhat or very important. Participants were also asked questions about compensation for their participation (Fig. 1). Seventy-four percent of all participants felt that compensation was adequate. Fifty-one percent of all participants indicated that compensation influenced their attendance at study visits. One participant felt that decreases in compensation caused them to miss study visits.

### Opinions and experience with Truvada

Participants were asked several questions about Truvada®. Twenty-six (60%) participants had previously been on Truvada® for pre-exposure prophylaxis and 15 (58%) participants had received Truvada® through a research study. Ninety-five percent of all participants indicated that they were likely to participate in a research study if Truvada® was provided.

### Experience with research staff and site

**Fig. 2:**
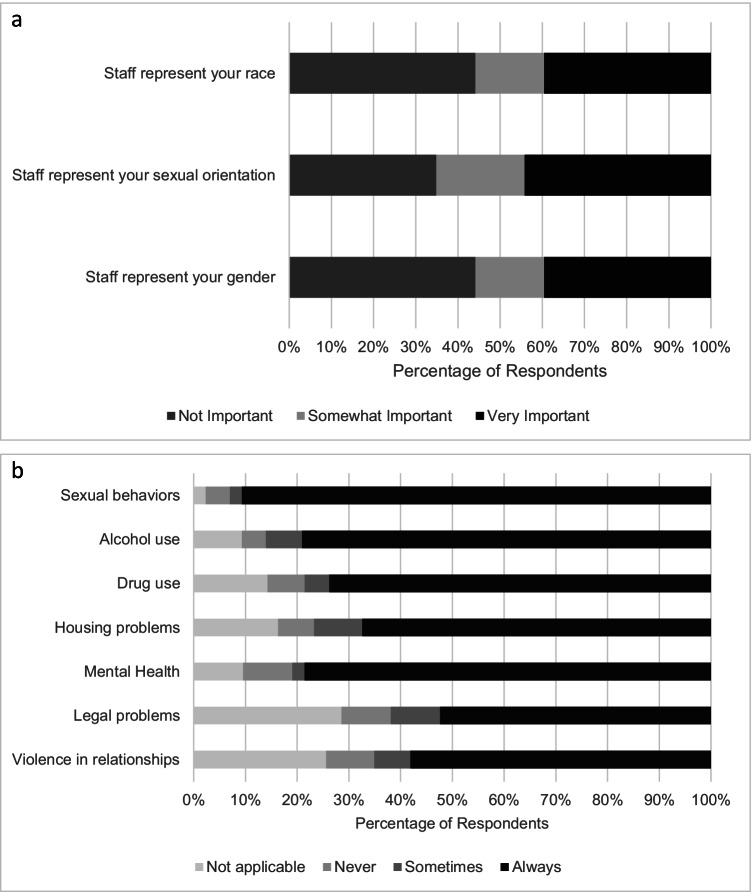
Participants’ attitudes regarding research staff identity congruence and comfort discussing sensitive topics. **a** Participants were asked to indicate the importance of research staff sharing similar identities to them for their willingness to participate in research. **b** Participants were asked to indicate how frequently research staff tried to make them feel comfortable when discussing sensitive topics

Participants were asked about their interactions with research staff. Ease of contact with research staff was rated on a 5-point Likert scale (easy to difficult). Thirty-six (84%) participants indicated that it was somewhat easy or easy to get in touch with the research team when they needed. Participants were also asked to indicate the importance of staff representing their gender, sexual orientation, and race using a 3-point Likert scale (not important, somewhat important, very important) (Fig. 2a). Twenty-one (65%) participants felt that it was somewhat or very important that staff represent their sexual orientation, 24 (56%) participants felt that it was somewhat or very important that staff represent their race, and 24 (56%) participants felt that it was somewhat or very important that staff represent their gender. Participants rated their comfort with discussing sensitive topics with research staff during their risk reduction counseling sessions (Fig. 2b). Participants reported that research staff sometimes or always made them feel comfortable when talking about sexual behaviors (95%, *n* = 40), alcohol use (95%, *n* = 37), mental health (89%, *n* = 34), drug use (92%, *n* = 33), housing problems (92%, *n* = 33), violence in relationships (88%, *n* = 28), and legal problems (87%, *n* = 26).

### Utilization of ancillary resources

NJMS CRC provides non-study-specific ancillary resources in the research space such as a condom dispenser, event fliers, and a community resource board. The site also hosts multiple community engagement events. Participants’ utilization and attendance at these events was assessed. Thirty-four (77%) participants said that they used at least one resource in the space, with the condom dispenser being most used. The most frequently cited event that participants attended was the annual holiday dinner which is held in December at a local restaurant to express our appreciation to participants for their continued engagement in research and the mission of the NJMS CRC; 28 (64%) participants attended.

### Barriers to research participation

Participants were asked about issues that would potentially prevent them from continued participation in the HIV prevention research study over the next year. Participants could select more than one potential issue. Seventeen (39%) participants reported their job or school schedule, 8 (18%) participants reported possible relocation, 5 (11%) participants reported reimbursement, and 4 (9%) participants reported transportation issues. Twenty (45%) participants indicated that they did not anticipate any problems with continued study participation. Additionally, barriers relating to support, including resistance from partners or family, lack of social support, and disapproval from support systems, were not reported as significant barriers to continued study participation.

### Overall satisfaction

Participants were asked to rate their overall research experience. When asked whether their opinion of the study changed once they had started the study, 32 (74%) participants said that they liked the study more, 1 (2%) participant said that they liked the study less, and 10 (23%) participants said that their overall opinion did not change. Forty-two (98%) participants rated their experience as good or very good, 39 (91%) participants said that they would participate in future research studies at the research site, and 41 (95%) participants indicated that they would recommend the center’s research studies and services to a friend.

## Discussion

In this study of the perspectives of cisgender MSM and transgender persons participating in HIV prevention research at our site, 70% of respondents were Black and Latinx MSM or transgender POC and participants reported high levels of satisfaction with their experience participating in HIV prevention research studies. Most respondents indicated that they viewed core study responsibilities, like receiving injections or taking pills, as somewhat or very important, and additionally reported feeling satisfied with the study visit length, time commitments, and compensation. Participants reported feeling comfortable discussing potentially sensitive topics with staff and the vast majority indicated that they would participate in future research studies and/or refer friends for participation. There were no statistically significant differences in responses by participants’ sociodemographic characteristics. Prior studies of Black and Latinx MSM and transgender POC have documented multiple barriers to clinical trials participation [2, 12–14]. The most commonly cited barriers are mistrust of health care providers and researchers, provider bias, past negative experiences with the healthcare system, and stigma of being perceived as living with HIV [12–14]. Additionally, logistical barriers such as inadequate transportation and insufficient incentives are impediments to participation [2, 12–14]. Our participants may not have reported these barriers as they had participated in National Institutes of Health sponsored HIV prevention trials and there has been concerted efforts to develop studies that consider the interests and needs of these communities when designing the trials [13, 15]. At a local level, we employed strategies to build community partnerships and develop cross-cultural competence among our team [12, 14, 16, 17].

Regarding barriers to research participation, our survey participants reported that job or school schedules and possible relocation would prevent them from participating in future research. Other studies have found that conflict with work and life schedules is an important barrier to participation [12, 13]. It has been found that higher levels of education are generally associated with willingness to participate in research [18]. In a study of barriers and facilitators of HIV vaccine and prevention study participation among young non-Hispanic Black and Hispanic Black MSM and transwomen, education levels were positively associated with a willingness to participate in HIV vaccine trials in the univariate analysis, but became non-significant in the multivariate analysis [2]. Our study did not determine if it was job, schooling or both that were potential barriers to future research participation, and our findings were based on a non-representative, small convenience sample that limits any conclusions about the influence of education on research participation for Black and Latinx cisgender MSM and transgender POC. Future research in this area should further evaluate education levels as a potential barrier and consider the needs of students participating in research in the study design.

Resistance from partners or family, lack of social support, and disapproval from support systems have been cited as potential barriers to research participation for Black and Latinx cisgender MSM and transgender POC, but these factors were not reported as significant barriers to study participation among participants in our study [2]. Additionally, a lack of racial/ethnic, gender, and sexual identity congruence of research study staff with participants has been cited as adversely affecting research participant experiences [13, 19, 20]. Although 56–65% of participants indicated that it was important that staff represent their gender, race or ethnicity, or sexual orientation, over 85% of participants felt comfortable discussing sensitive topics with the research team which included discussions about sexual behaviors, drug use, intimate partner violence, and legal problems. This finding may be due to high quality of interactions with site staff, who regularly undergo training in cultural competency and gender-affirming care [13, 15, 21].

There were several limitations to this research study. The sample size was small due to a low response rate of 37.6% and this may have been due to recruitment challenges related to the onset of coronavirus 2019 pandemic. The sample was also primarily of cisgender MSM and therefore has limited representation of transgender participants’ research perspectives. This study may also suffer from non-response bias in which participants who did not respond to the survey were more likely to be dissatisfied with the HIV prevention study. Finally, there is a lack of a validated survey to assess barriers and facilitators to participation in HIV prevention research.

The numerous studies of the attitudes of Black and Latinx MSM and transgender POC about biomedical interventions for HIV prevention indicate a generally high interest in these modalities and a desire for a plurality of options to allow for the development of a tailored HIV prevention plan [19, 20, 22–24]. However, the representation of these communities in clinical trials remains suboptimal. Research on the barriers and facilitators to clinical trials participation for HIV prevention among Black and Latinx MSM and transgender POC is still an emerging field which is particularly lacking in studies that focus solely or predominantly on the perspectives of Latinx or other POC. The barriers to inclusive research participation are not insurmountable and our study illustrates that carefully constructing studies and research environments that are welcoming, safe, and responsive to the concerns of Black and Latinx MSM and transgender POC can effectively overcome many barriers to the recruitment and retention of these communities into HIV prevention research.

## Conclusions

Engaging key communities in HIV prevention and clinical research trials is critical to putting an end to the HIV epidemic. Involving community members in the initial design and conductance of the study will help ensure a satisfactory trial experience for research participants and foster a long-standing research-community partnership. Conducting thorough post-trial evaluations and assessing participant experiences with biomedical interventions, trial responsibilities, and the research site and staff contributes to data-driven quality control and improvement of future clinical trial recruitment and retention.

## References

[1] Centers for Disease Control and Prevention. HIV Surveillance Report, 2019. 2021 May. Report No.: vol, 32. Available from: http://www.cdc.gov/hiv/library/reports/hiv-surveillance.html

[2] Richardson S, Seekaew P, Koblin B, Vazquez T, Nandi V, Tieu H-V (2017). Barriers and facilitators of HIV vaccine and prevention study participation among Young Black MSM and transwomen in New York City. PLoS ONE.

[3] Huamani KF, Metch B, Broder G, Andrasik M (2019). A Demographic analysis of racial/ethnic minority enrollment into HVTN preventive early phase HIV vaccine clinical trials conducted in the United States, 2002–2016. Public Health Rep.

[4] Djomand G, Katzman J, di Tommaso D, Hudgens MG, Counts GW, Koblin BA (2005). Enrollment of racial/ethnic minorities in NIAID-funded networks of HIV vaccine trials in the United States, 1988 to 2002. Public Health Rep.

[5] Castillo-Mancilla JR, Cohn SE, Krishnan S, Cespedes M, Floris-Moore M, Schulte G (2014). Minorities Remain underrepresented in HIV/AIDS research despite access to clinical trials. HIV Clin Trials.

[6] Tross S, Pinho V, Lima JE, Ghiroli M, Elkington KS, Strauss DH (2018). Participation in HIV behavioral research: unanticipated benefits and burdens. AIDS Behav.

[7] Odero I, Ondeng’e K, Mudhune V, Okola P, Oruko J, Otieno G (2019). Participant satisfaction with clinical trial experience and post-trial transitioning to HIV care in Kenya. Int J STD AIDS..

[8] Murray MI, Markowitz M, Frank I, Grant RM, Mayer KH, Hudson KJ (2018). Satisfaction and acceptability of cabotegravir long-acting injectable suspension for prevention of HIV: Patient perspectives from the ECLAIR trial. HIV Clin Trials.

[9] Kerrigan D, Mantsios A, Grant R, Markowitz M, Defechereux P, La Mar M (2018). Expanding the menu of hiv prevention options: a qualitative study of experiences with long-acting injectable cabotegravir as PrEP in the context of a Phase II trial in the United States. AIDS Behav.

[10] Harris PA, Taylor R, Thielke R, Payne J, Gonzalez N, Conde JG (2009). Research electronic data capture (REDCap)—a metadata-driven methodology and workflow process for providing translational research informatics support. J Biomed Inform.

[11] Harris PA, Taylor R, Minor BL, Elliott V, Fernandez M, O’Neal L (2019). The REDCap consortium: Building an international community of software platform partners. J Biomed Inform.

[12] Reisner SL, Chaudhry A, Cooney E, Garrison-Desany H, Juarez-Chavez E, Wirtz AL (2020). ‘It all dials back to safety’: a qualitative study of social and economic vulnerabilities among transgender women participating in HIV research in the USA. BMJ Open.

[13] Watson CC, Wilton L, Lucas JP, Bryant L, Victorianne GD, Aradhya K (2020). Development of a black caucus within the HIV prevention trials network (HPTN): representing the perspectives of black men who have sex with men (MSM). Int J Environ Res Public Health.

[14] Yoon R, Mooney J, Broder G, Bolton M, Votto T, Davis-Vogel A (2014). Exploring barriers and facilitators to participation of male-to-female transgender persons in preventive HIV vaccine Clinical Trials. Prev Sci.

[15] Siskind RL, Andrasik M, Karuna ST, Broder GB, Collins C, Liu A (2016). Engaging transgender people in NIH-funded HIV/AIDS clinical trials research. J Acquir Immune Defic Syndr.

[16] Israel BA, Schulz AJ, Parker EA, Becker AB (1998). Review of community-based research: assessing partnership approaches to improve public health. Annu Rev Public Health.

[17] Alio AP, Sibley C, Ouedraogo HS, Wallace SE, Wakefield S, Humes DL (2020). House ball community leaders’ perceptions of HIV and HIV vaccine research. Int J MCH AIDS.

[18] Walter JK, Davis MM (2016). Who’s Willing? Characteristics associated with willingness to participate in clinical research. IRB.

[19] Sevelius JM, Keatley J, Calma N, Arnold E (2016). “I am not a man”: Trans-specific barriers and facilitators to PrEP acceptability among transgender women. Glob Public Health.

[20] Biello KB, Hosek S, Drucker MT, Belzer M, Mimiaga MJ, Marrow E (2018). Preferences for Injectable PrEP Among Young U.S. Cisgender men and transgender women and men who have sex with men. Arch Sex Behav..

[21] Asquith A, Sava L, Harris AB, Radix AE, Pardee DJ, Reisner SL (2021). Patient-centered practices for engaging transgender and gender diverse patients in clinical research studies. BMC Med Res Methodol.

[22] Mansergh G, Kota KK, Stephenson R, Hirshfield S, Sullivan P (2021). Preference for using a variety of future HIV pre-exposure prophylaxis products among men who have sex with men in three US cities. J Int AIDS Soc.

[23] Rael CT, Martinez M, Giguere R, Bockting W, MacCrate C, Mellman W (2020). Transgender women’s concerns and preferences on potential future long-acting biomedical HIV Prevention strategies: the case of injections and implanted medication delivery devices (IMDDs). AIDS Behav.

[24] Rael CT, Lopez-Ríos J, McKenna SA, Das D, Dolezal C, Abascal E (2021). Transgender Women’s barriers, facilitators, and preferences on tailored injection delivery strategies to administer long-acting injectable cabotegravir (CAB-LA) for HIV pre-exposure prophylaxis (PrEP). AIDS Behav.

